# *IRX3* Promotes the Browning of White Adipocytes and Its Rare Variants are Associated with Human Obesity Risk

**DOI:** 10.1016/j.ebiom.2017.09.010

**Published:** 2017-09-13

**Authors:** Yaoyu Zou, Peng Lu, Juan Shi, Wen Liu, Minglan Yang, Shaoqian Zhao, Na Chen, Maopei Chen, Yingkai Sun, Aibo Gao, Qingbo Chen, Zhiguo Zhang, Qinyun Ma, Tinglu Ning, Xiayang Ying, Jiabin Jin, Xiaxing Deng, Baiyong Shen, Yifei Zhang, Bo Yuan, Sophie Kauderer, Simin Liu, Jie Hong, Ruixin Liu, Guang Ning, Weiqing Wang, Weiqiong Gu, Jiqiu Wang

**Affiliations:** a Shanghai Institutes for Biological Sciences, University of Chinese Academy of Sciences, Chinese Academy of Sciences, Shanghai 200031, China; b Department of Endocrinology and Metabolism, National Key Laboratory for Medical Genomes, China National Research Center for Metabolic Diseases, Ruijin Hospital, Shanghai Jiao Tong University School of Medicine (SJTUSM), Shanghai 200025, China; c Shanghai Ji Ai Genetics & IVF Institute, Obstetrics & Gynecology Hospital, Fudan University, Shanghai 200011, China; d Pancreatic Disease Center, Department of General Surgery, Ruijin Hospital, SJTUSM, Shanghai 200025, China; e Department of Burns and Plastic Surgery, Ruijin Hospital, SJTUSM, Shanghai 200025, China; f Department of Epidemiology and Center for Global Cardiometabolic Health, School of Public Health, Department of Medicine (Endocrinology), Warren Alpert Medical School, Brown University, Providence, RI 02912, USA

**Keywords:** IRX3, Beige adipocytes, Browning program, Genetic variants, Obesity

## Abstract

**Background:**

IRX3 was recently reported as the effector of the *FTO* variants. We aimed to test IRX3's roles in the browning program and to evaluate the association between the genetic variants in *IRX3* and human obesity.

**Methods:**

IRX3 expression was examined in beige adipocytes in human and mouse models, and further validated in induced beige adipocytes. The browning capacity of primary preadipocytes was assessed with IRX3 knockdown. Luciferase reporter analysis and ChIP assay were applied to investigate IRX3's effects on *UCP1* transcriptional activity. Moreover, genetic analysis of *IRX3* was performed in 861 young obese subjects and 916 controls.

**Results:**

IRX3 expression was induced in the browning process and was positively correlated with the browning markers. IRX3 knockdown remarkably inhibited UCP1 expression in induced mouse and human beige adipocytes, and also repressed the uncoupled oxygen consumption rate. Further, IRX3 directly bound to *UCP1* promoter and increased its transcriptional activity. Moreover, 17 rare heterozygous missense/frameshift *IRX3* variants were identified, with a significant enrichment in obese subjects (*P* = 0.038, OR = 2.27; 95% CI, 1.02–5.05).

**Conclusions:**

*IRX3* deficiency repressed the browning program of white adipocytes partially by regulating *UCP1* transcriptional activity. Rare variants of *IRX3* were associated with human obesity.

## Highlights

• IRX3 expression was positively correlated with the browning markers in both mice and humans.
• IRX3 deficiency repressed the browning program in human and mouse cell models by regulating UCP1 transcriptional activity.
• Rare variants of IRX3 were associated with human obesity.

While FTO is the first risk gene for obesity identified via genome-wide association studies, its exact mechanism remains unknown until two recent studies reported that IRX3, as FTO's target, probably inhibited the browning of white fat. However, whether IRX3 gene per se predisposes to obesity in humans and how IRX3 regulates the browning process remains unclear. Herein we combined cellular and genetic evidence to support that IRX3 promotes but not inhibit the browning process in both human and mouse cell models. Mechanistically, IRX3 increased the transcriptional activity of UCP1 through directly binding to its promoter. Thus, our findings provided an intact picture of IRX3's function in browning program and human obesity.

## 1 Introduction

Obesity is a complex disease determined by genetic and environmental factors. The heritability of obesity is as high as 40–70% (Stunkard et al., 1990; Allison et al., 1996), which makes decipherment of obese-related genes extremely important for obesity treatment. The strongest obesity-associated loci were consistently identified as located in the first and second introns of *FTO* (Scuteri et al., 2007; Dina et al., 2007; Frayling et al., 2007). However, the direct causality between *FTO* and obesity has not been clearly validated. Recently, two studies made a breakthrough in identifying IRX3 as the target of the risk loci in the *FTO* gene for human obesity (Smemo et al., 2014; Claussnitzer et al., 2015). One study showed the regulatory role of *IRX3* in the browning of white adipose tissue (WAT) and obesity development through hypothalamus-specific (*Insulin2*-cre) *Irx3* knockout mouse models (Smemo et al., 2014), while the other indicated that the *FTO* obesity-linked loci functioned as a body weight regulator by controlling IRX3 expression in the peripheral adipose tissue and IRX3 modulated the browning progress of WAT, which was further supported by adipose-specific (*Ap2*-cre) *Irx3* knockout mice (Claussnitzer et al., 2015). However, the detailed effects of *IRX3* on browning shift of white adipocytes remains undetermined.

Generation of inducible browning adipocytes (also called brown-like/beige adipocytes) confers beneficial effects against adiposity (Virtanen et al., 2009; Wu et al., 2012; Cypess et al., 2009). Beige adipocytes specialize in heat production by consuming a large amount of fatty acids and glucose, thus promoting energy expenditure. As a growing body of studies has demonstrated the existence of beige adipocytes in infants and adults (Cypess et al., 2009, 2013; Lidell et al., 2013) and its close association with reduced body weight has been discovered (Guerra et al., 1998), the browning program of WAT becomes an intriguing target to combat obesity and its metabolic consequences.

*IRX3*, a transcription factor belonging to the Iroquois family of homeobox genes, participates in the pattern and development of several tissues, including the central nervous system (Bellefroid et al., 1998), microvascular system (Scarlett et al., 2015), kidney (Reggiani et al., 2007), and heart (Zhang et al., 2011). Two heterozygous *IRX3* mutations have been linked to idiopathic ventricular fibrillation in humans (Koizumi et al., 2015), suggesting that nonsynonymous variant in *IRX3* could be functional and lead to certain diseases. With regard to the close association of *IRX3* with the *FTO* obesity-linked loci, revealing *IRX3*'s genetic profile can be of great importance to explain how genetic factors manipulate body weight alteration. However, whether the genetic variants of *IRX3* are associated with human obesity and how *IRX3* regulates the browning process remain unclear.

In the present study, we aimed to clarify the direct role and possible mechanism of *IRX3* in the browning program of white adipocytes by using diverse browning models and in vitro experiments. We found that *Irx3* disruption dramatically inhibited the browning program of preadipocytes, showing reduced Ucp1 expression and uncoupled oxygen consumption, and the effects might be achieved at least partially through inhibiting *Ucp1* transcriptional activity. Targeted exons sequencing methods have been widely and successfully used to explore functional rare variants in relation to obesity and type 2 diabetes (Pearce et al., 2013; Ramachandrappa et al., 2013; Flannick et al., 2014; Bonnefond et al., 2012). Therefore, we further conducted targeted sequencing of *IRX3* coding sequence in the lean and young obese subjects and found that rare *IRX3* variants were significantly associated with human obesity. These findings provide more extensive information regarding the regulatory role of *IRX3* in the browning process and human obesity.

## 2 Methods

### 2.1 Mouse Adipose Tissues

Male C57BL/6J mice at eight weeks old were subjected to cold room (4 °C) stress for one week (Wang et al., 2013) or received daily intraperitoneal injections of CL-316,243 (1 mg/kg, Sigma-Aldrich, C5976) for 10 days as described previously (Dempersmier et al., 2015). The dissected tissues were rapidly frozen and stored in liquid nitrogen. All the animal experiments were approved by the Animal Care Committee of Shanghai Jiao Tong University School of Medicine.

### 2.2 RNA Extraction and Real-Time PCR

Samples were homogenized with TRIzol reagent (Thermo Fisher Scientific Inc., 15596018) and total RNA was extracted according to the manufacturer's instructions. 1 μg of RNA was reverse transcribed to cDNA with a reverse transcription kit (Promega, A3500). Real-time PCR was carried out on the QuantStudio Dx system (Thermo Fisher Scientific Inc.) using SYBR Green Supermix (Takara Bio, DRR041A). The primers used in this study are provided in Table S1. Quantitative amounts of gene expression were normalized to the housekeeping gene mouse *36b4* or human *βactin* and analyzed using the 2^− ΔΔCT^ methods. The results are representative of at least three independent experiments.

### 2.3 Western Blotting

Western blotting (WB) was performed as previously described (Wang et al., 2013). Proteins were assessed with the following antibodies: anti-IRX3 antibody (Abcam, ab25703), anti-GFP antibody (Cell Signaling Technology, 2956s), anti-actin antibody (Santa Cruz Biotechnology, sc-8432), anti-UCP1 antibody (Alpha Diagnostic, ucp1-a), anti-HSP90 antibody (Cell Signaling Technology, 4877s), anti-PGC-1α antibody (Millipore, ab3242), anti-AP2 antibody (Cell Signaling Technology, 3544s), and horseradish peroxidase-conjugated (HRP)-linked secondary antibody (Cell Signaling Technology, 7076, 7074). The representative blotting bands were repeated in at least three mice. The results are representative of at least three independent experiments.

### 2.4 Morphological Studies

Adipose tissues were stained with hematoxylin and eosin (HE) according to the standard protocols. For immunohistochemistry, rabbit anti-UCP1 antibody (Alpha Diagnostic, ucp1-a, diluted 1:400) or rabbit anti-IRX3 (Abcam, ab25703, diluted 1:1000) was used to incubate the sections, followed by incubation with HRP-linked secondary antibody (Dako, k500711). Electron microscopy of browning adipose tissue was performed as previously described (Wang et al., 2013). To determine the localization of IRX3 in the browning adipocytes, beige cells after eight days induction were fixed and incubated with rabbit anti-IRX3 antibody (Abcam, ab25703) and mouse anti-UCP1 antibody (R&D, MAB6158), followed by incubation with Alexa Fluor 594-conjugated goat anti-rabbit secondary antibody (ThermoFisher, R37117), 488–conjugated goat anti-mouse secondary antibody (ThermoFisher, A32723), and DAPI (SouthernBiotech, 0110-20). Images were acquired with a confocal laser-scanning microscope (Zeiss). All the images were representative of three independent experiments.

### 2.5 Human Adipose Tissue Samples

Human browning omental adipose tissue for immunohistochemistry was obtained from clinically and pathologically diagnosed patients with pheochromocytoma and sex-, age-, and BMI-matched control subjects. Primary human preadipocytes in stromal vascular fraction (SVF) from subcutaneous WAT (sWAT) for the induction to beige adipocytes were obtained from an underaged subjects. The biopsy samples of abdominal sWAT and omental WAT (oWAT) for the comparison of IRX3 expression were obtained from 21 severely obese subjects and 9 lean subjects. Obese patients with secondary or syndromic obesity-related diseases were excluded. Detailed characteristics of the subjects are presented in Table S2. The adipose tissue pieces were obtained during surgical procedures followed by immediate storage in liquid nitrogen and fixation in formalin. The human study was approved by the Institutional Review Board of Ruijin Hospital, Shanghai Jiao Tong University School of Medicine. Written informed consent was provided from each participant prior to inclusion in the study.

### 2.6 Stromal Vascular Fraction Isolation, Beige/Brown and White Adipocyte Differentiation

SVFs were isolated from the inguinal WAT (IWAT), epididymal WAT (EWAT) or brown adipose tissue (BAT) of male mice with C57BL/6J or 129/Sv background and induced into fully differentiated beige/brown adipocytes or white adipocytes as previously described (Wang et al., 2013). Briefly, the adipose tissue was minced and then digested with type II collagenase (Sigma) in 10 mM HEPES (Sigma) for 30 min at 37 °C. Digested adipose tissue was filtered through a 40 μm cell strainer to remove large undigested pieces. After centrifuge for 10 min, SVF cells were then resuspended with DMEM/F12 medium. For the induction of beige/brown adipocytes, SVF cells were plated onto 48-well plates to reach confluence and incubated with medium supplemented with 5 μg/ml insulin (Eli Lilly, HI0240), 0.5 mM isobutylmethylxanthine (Sigma-Aldrich, I7018), 1 μM dexamethasone (Sigma-Aldrich, D4902), 1 nM T3 (Sigma-Aldrich, T2877), and 1 μM rosiglitazone (Sigma-Aldrich, R2408) for four days, and subsequently in medium with insulin, T3, and for another four days. For the induction to white adipocytes, cells were incubated with medium supplemented with 5 μg/ml insulin, 0.5 mM isobutylmethylxanthine, and 1 μM dexamethasone for two days, and subsequently in medium with insulin 5 μg/ml insulin for six days.

### 2.7 Cell Culture

SVFs from murine or human were cultured in DMEM/F12 supplemented with 10% FBS, 1% P/S, 1 mM L-glutamine, and 10 ng/ml murine basic fibroblast growth factor (R&D Systems, 3139-FB-025). HEK293T cells were purchased from ATCC.

### 2.8 Lentiviral shRNA Generation

Lentiviral mouse *Irx3* shRNA constructs and lentiviral human *IRX3* constructs were made using pLKO-PGK-RFP and pLKO-CMV-RFP lentivector cloning, respectively. We verified all constructs using DNA sequencing; the sequences of mouse *Irx3* shRNA were ccctatccaatgtgctttcat for *Irx3* shRNA-1 and tctgtgtatgattaccttaaa for *Irx3* shRNA-2. The sequences for human *IRX3* shRNA were ttgtaagcatgtccgtgtata for *hIRX3* shRNA-1 and gtttgtttgtccggttgattt for *hIRX3* shRNA-2. The cells were infected with the lentivirus to knockdown Irx3/IRX3 24 h after seeding.

### 2.9 Oil Red O Staining

After induction to mature adipocytes for eight days, mature adipocytes underwent Oil Red O staining as previously described (Wang et al., 2013). In brief, the cells were fixed with 4% paraformaldehyde for 30 min, fully rinsed, air dried, and incubated with Oil Red O (Sigma-Aldrich) for 30 min. The images were observed directly with microscope (Olympus).

### 2.10 Triglyceride Measurement

After the induction for 8 days, mature beige adipocytes were collected for triglyceride (TG) measurement guided by the manufacturer's protocols (BioVision, K622-100).

### 2.11 Oxygen Consumption Rate Measurements

SVFs were seeded in an XF24 V28 microplate (Seahorse Bioscience) coated with poly-L-lysine. After 24 h, the cells were infected with lentivirus and subsequently differentiated into beige adipocytes for five days. Oxygen Consumption Rate (OCR) was measured as previously described (Wang et al., 2013) using an XF24 analyzer (Seahorse Bioscience) in accordance with the manufacturer's instructions. In brief, the cells were washed with Seahorse XF base medium with 25 mM D-glucose, 2 mM sodium pyruvate, and 2 mM LG, followed by incubation with 525 μl assay medium with or without 2% fatty acid-free BSA at 37 °C in a non-CO_2_ incubator (Seahorse Bioscience) for 1 h. 75 μl respiratory inhibitors (Seahorse Bioscience, 103015-100) was loaded into the injection port to reach the final concentration of 1 mg/ml oligomycin, 1.5 mM FCCP, 0.5 mM antimycin A and 0.5 mg/ml rotenone to detect the uncoupled respiration, maximal respiration, and non-mitochondrial respiration, respectively. The final OCR results were standardized to the protein concentration. The results are representative of at least three independent experiments.

### 2.12 Plasmid Construction

The coding region of *IRX3* PCR amplified from a human cDNA clone (NM_0243362) was sub-cloned into the pEGFP-C1 vector (N-terminal GFP-tag) between the *Hin*dIII and *Bam*HI restriction sites. Plasmids of murine *Ucp1* promoter and human *UCP1* promoter, which was 4 kb proximal to the transcriptional start site (TSS), was constructed into the pGL-4.12 vector (Promega) as previously described (Zhang et al., 2014). Mutagenesis was generated using a site-directed mutagenesis kit (Agilent Technologies). Full-length coding sequences for all the plasmids were verified by DNA sequencing.

### 2.13 Luciferase Reporter Assay

Luciferase reporter assay was conducted in HEK293T cells. After 48 h transfection with 800 ng pEGFP-C1 vector or pEGFP-C1-IRX3 plasmids, along with 1 ng pRL-TK (or alternatively 1 ng pRL-SV40) and 200 ng *UCP1/Ucp1* promoter reporter construct, HEK293T cells were harvested for luciferase activity assessment using a dual-luciferase reporter assay system (Promega). The final results were normalized to Renilla luciferase activity. The results are representative of at least three independent experiments.

### 2.14 Chromatin Immunoprecipitation Assay

Chromatin immunoprecipitation (ChIP) assay was carried out to investigate the interaction between IRX3 and the *Ucp1* promoter using a commercial kit (Millipore, 17–371) in accordance with the manufacturer's instructions. Nuclei were extracted from the mature beige adipocytes induced from preadipocytes within IWAT of C57BL/6J mice. For immunoprecipitation, equal aliquots of cell lysates were incubated with anti-IRX3 antibody or IgG overnight at 4 °C. The precipitated DNA fragments were analyzed by real-time PCR with the following mouse *Ucp1* primers: forward primer 5′-GAGTAGCCGAAGGGTTCAGG-3′; reverse primer 5′-TCCCATAGGAAGCGTCATGC-3′′.

### 2.15 Participants in Case-Control Genetic Study

In stage one, we used an in-home whole-exome sequencing database that included 227 young obese subjects and 219 lean controls from eastern China as described in our recent study (Liu et al., 2017; Zou et al., 2017). It has been established that the population shows high genetic homogeneity to minimize any effects of population stratification (Liu et al., 2017). All the patients were continuously recruited from 2007 to 2011 from the specialized obesity outpatient clinic of Ruijin Hospital, Shanghai Jiao Tong University School of Medicine. Participants were excluded if they had secondary or syndromatic obesity; previous pharmacological, diet, or exercise intervention; infectious disease, hepatic or renal dysfunction, or malignant tumors. Trained physicians used height-weight scales to take measurements of the height and weight three times for each subject; mean values of each variable were used for further analysis. Genetic and basic characteristic information were obtained from the Genetics of Obesity in Chinese Youngs (GOCY) study (Wang et al., 2013; Liu et al., 2017; Zou et al., 2017; Hong et al., 2013) that was previously established by Ruijin Hospital and registered in ClinicalTrials.gov. In stage two, 634 young obese patients and 697 controls were additionally recruited into the study according to the same procedure. This study was also approved by the Institutional Review Board of Ruijin Hospital, Shanghai Jiao Tong University School of Medicine. Written informed consent was obtained from each participant.

### 2.16 Genotyping

Genomic DNA was isolated from peripheral blood using a commercial blood genomic DNA extraction kit (Qiagen). Sanger sequencing was performed to identify nonsynonymous variants, including frameshift, nonsense, deletion, or missense mutations in *IRX*3 (Gene ID: 79,191) exons with minor allele frequency (MAF) < 1%. Primers used to amplify the *IRX3* exons are shown in Table S3.

### 2.17 Sequence Alignment of *IRX3* Orthologs Related to the Studied Variants

Sequence alignment of IRX3 across different species related to the variants identified in obese subjects and controls was conducted using a ClustalW2 alignment program (http://www.ebi.ac.uk/Tools/msa/clustalw2/). The comparison included the IRX3 amino acid sequence of human (*Homo sapiens*, NP_077312.2), mouse (*Mus musculus*, NP_032419.2), rat (*Rattus norvegicus*, NP_001100883.1), chimpanzee (*Pan troglodytes*, XP_510969.2), and dog (*Canis lupus familiaris*, XP_544403.2).

### 2.18 Statistical Analyses

Spearman's correlation analysis was performed to examine the associations between the expression of *Irx3* and brown adipocyte-related genes. Differences of the frequency distribution of rare *IRX3* variants between case patients and control subjects were calculated using the χ^2^ test. Unless otherwise stated, data was shown as mean ± S.E.M, and the results were compared by 2-tailed *t*-tests with α level of 0.05. *P* values < 0.05 were considered to be significantly different. As to basic experiments, data were generated from three independent experiments. Analyses were carried out with SAS version 9.3 (SAS Institute, New York, NY, USA).

## 3 Result

### 3.1 *IRX3* was Induced in the Browning Program of WATs in Both Mice and Humans

**Fig. 1:**
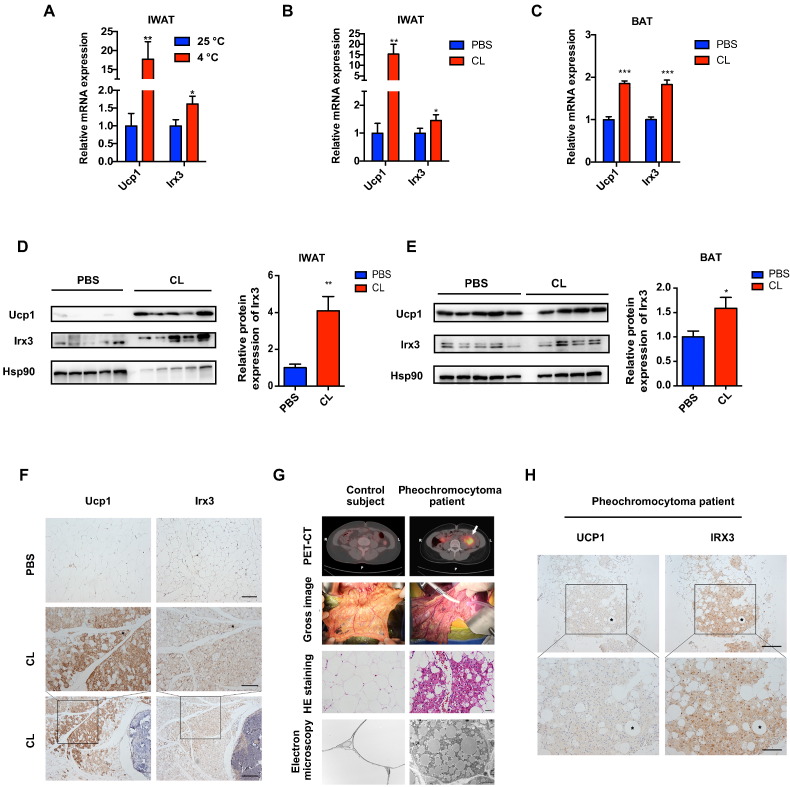
IRX3/Irx3 expression is induced in browning adipose tissues. (A) Relative mRNA levels of *Ucp1* and *Irx3* in IWAT from mice under cold stress (4 °C) for one week (*n* = 6) and at 25 °C (*n* = 8). (B–C) Relative mRNA levels of *Ucp1* and *Irx3* in IWAT (B) and BAT (C) from mice subjected to PBS or CL-316,243 for 10 days. For A–C, gene expression was normalized to *36b4*. (D–E) Protein levels of Ucp1 and Irx3 (left) and the quantification value of Irx3 relative to Hsp90 (right) in IWAT (D) and BAT (E) in the mice subjected to PBS or CL-316,243 treatment. (F) Representative images of immunohistochemical staining in IWAT from mice subjected to PBS or CL-316,243 treatment (scale bar, 50 μm for the upper and middle panels, and 100 μm for the bottom panel). (G) PET-CT, gross image, HE staining (scale bar, 20 μm), and electron microscopy (scale bar, 5 μm) of the browning WAT from pheochromocytoma patients. (H) Representative images of immunohistochemical staining in oWAT from pheochromocytoma patients (scale bar, 100 μm for the upper panel, and 50 μm for the bottom panel). Data were presented as mean ± s.e.m. *P < 0.05, **P < 0.01, ***P < 0.001. The results are representative of at least three independent experiments. IWAT, inguinal white adipose tissue. BAT, brown adipose tissue.

To identify the involvement of *IRX3* in the generation of beige adipocytes within WAT, we first detected *IRX3* expression during the induction of the browning program. In rodents, the browning program of white fat depots can be activated by prolonged cold stress or β3-adrenergic agonist CL-316,243 (Rosen and Spiegelman, 2014; Himms-Hagen et al., 1994). We found that cold stress (4 °C) increased the mRNA levels of *Irx3* in IWAT coincident with the induction of brown adipocyte marker, *Ucp1* (Fig. 1A). We did not observe an increase in IRX3 protein levels under cold stress (Supplementary Fig. 1). Interestingly, a significantly higher mRNA and protein level of Irx3 was also observed in the IWAT and classical BAT of the mice injected with CL-316,243 than the control group, in parallel with the induction of *Ucp1* (Fig. 1B–F). Pheochromocytoma patients, characterized by excess β3-adrenergic activation, have abundant beige adipocytes (Puar et al., 2016). We therefore further validated *IRX3* expression in the beige adipocytes from pheochromocytoma patients. The browning fat of pheochromocytoma patients were confirmed by the enhanced ^18^F-2-deoxy-2-fluoro-d-glucose uptake by PET/CT scan, tissue morphology, increased mitochondria under electron microscopy, and positive UCP1 protein staining (Fig. 1G–H). Consistently, we found a positive staining of *IRX3* protein expression in the beige adipocytes (Fig. 1H). These results together suggested that *IRX3* expression is induced in the browning process of WATs in both mice and humans, especially under β3-adrenergic stimulation.

### 3.2 *IRX3* mRNA Levels were Positively Associated with Beige Adipocyte Markers

**Fig. 2:**
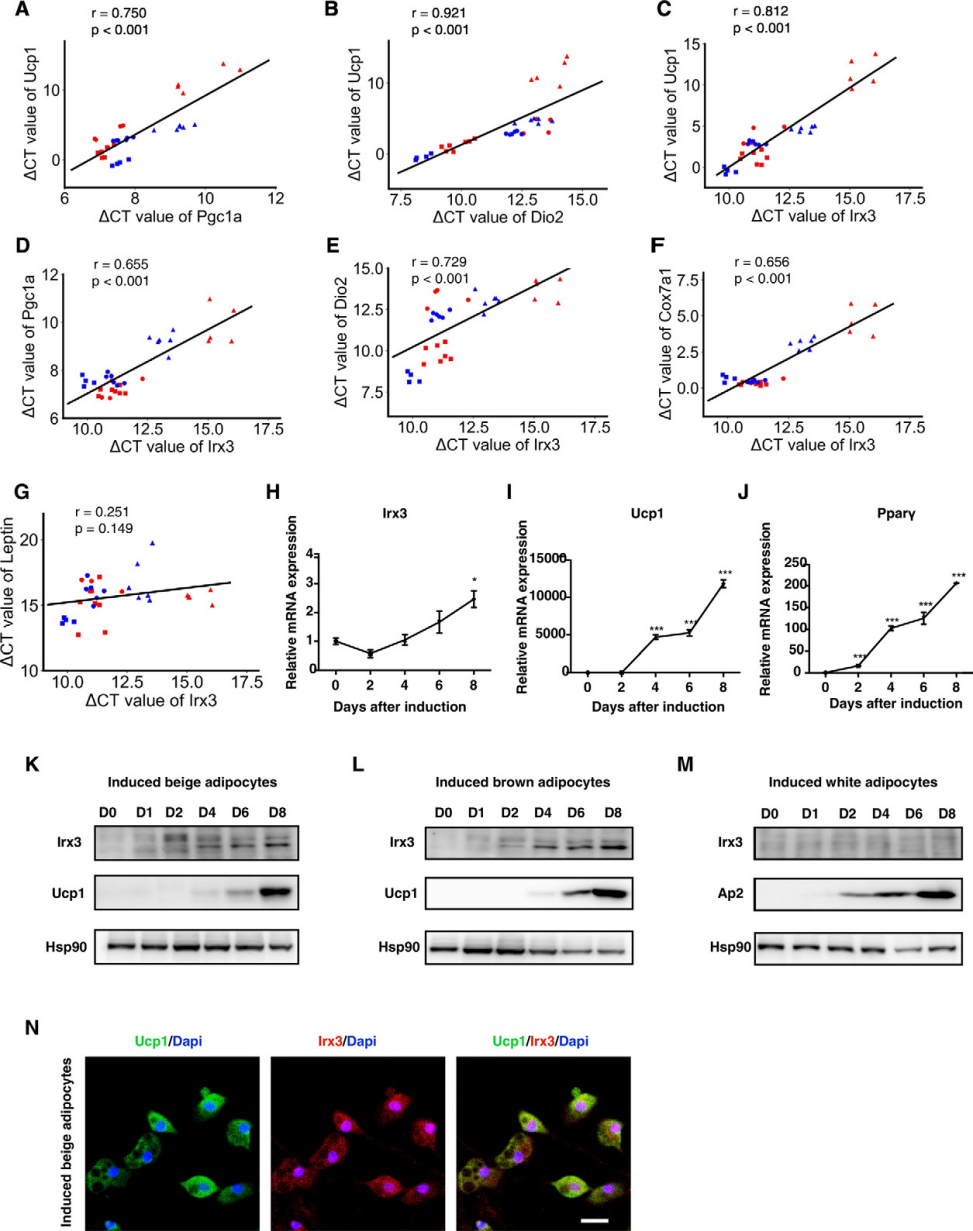
The expression of *Irx3* was correlated with the beige/brown adipocyte associated genes in vitro. (A–G) In fully differentiated beige/brown adipocytes from preadipocytes isolated from IWAT, EWAT, and BAT of C57BL/6J and 129/Sv mice, *Ucp1* mRNA expression was correlated with *Pgc-1α* (A) and *Dio2* (B). *Irx3* mRNA expression showed positive correlation with mRNA levels of (C) *Ucp1*, (D) *Pgc-1α*, (E) *Dio2*, and (F) *Cox7a1*. *Irx3* mRNA expression showed no correlation with *Leptin* (G). Cells from C57BL/6J were shown in red, and from 129/Sv were in blue. Cells from IWAT, EWAT, and BAT were shown as dot, triangle, and square, respectively. The correlation was analyzed using ΔCT. (H-J) (H) *Irx3* (I) *Ucp1* and (J) *Pparγ* mRNA expression during the time course of beige adipocyte differentiation cultures (*n* = 3). Quantitative amounts of gene expression were normalized to the housekeeping gene *36b4*. (K–L) Irx3 and Ucp1 protein levels at different time points during beige adipocyte differentiation from IWAT SVFs (K), during brown adipocyte differentiation from BAT SVFs (L). (M) Protein expression of Irx3 and Ap2 at different time points during white adipocyte differentiation from IWAT SVFs. (N) Intracellular location of Ucp1 and Irx3 protein in the fully differentiated beige adipocytes. Ucp1 protein, Irx3 protein and nucleus were indicated in green, red, and blue, respectively. Scar bar, 20 μm. Data were presented as mean ± s.e.m. *P < 0.05, **P < 0.01, ***P < 0.001. The results are representative of at least three independent experiments.

Primary preadipocytes residing in SVFs from EWAT, IWAT, and BAT, have the distinct capacity to differentiate into brown-like adipocytes (Macotela et al., 2012). We next isolated and differentiated SVFs from the three types of adipose tissues of two mouse strains with 129/Sv and C57BL/6J genetic backgrounds, respectively, to examine the correlation between the expression of *Irx3* and beige adipocyte markers. After an 8-day induction with beige/brown adipogenic procedures, *Ucp1* expression level was positively correlated with *Pgc-1α* and *Dio2* mRNA levels among different beige adipocytes (Fig. 2A-B). Concomitantly, *Irx3* mRNA levels presented a positive correlation with the expression of beige adipocyte-associated genes such as *Ucp1*, *Pgc-1α*, *Dio2*, and *Cox7a1* (Fig. 2C–F). No correlation between *Irx3* and white adipocyte marker *Leptin* was observed (Fig. 2G). We further detected Irx3 expression during the time course of beige/brown adipocyte differentiation. We found that Irx3 expression was increased during beige/brown adipocyte differentiation, in parallel with the induction of Ucp1 and Pparγ (Fig. 2H–L), while Irx3 expression was not induced during white adipocyte differentiation, as compared with the increase in Ap2 gene expression (Fig. 2M). Besides, Irx3 and Ucp1 were co-expressed in mature beige adipocytes (Fig. 2N). These findings further supported the possible promoting effects of *Irx3* on the browning program of white adipocytes.

### 3.3 *Irx3* Deficiency Repressed Beige Adipocyte Program and Biological Function in vitro

**Fig. 3:**
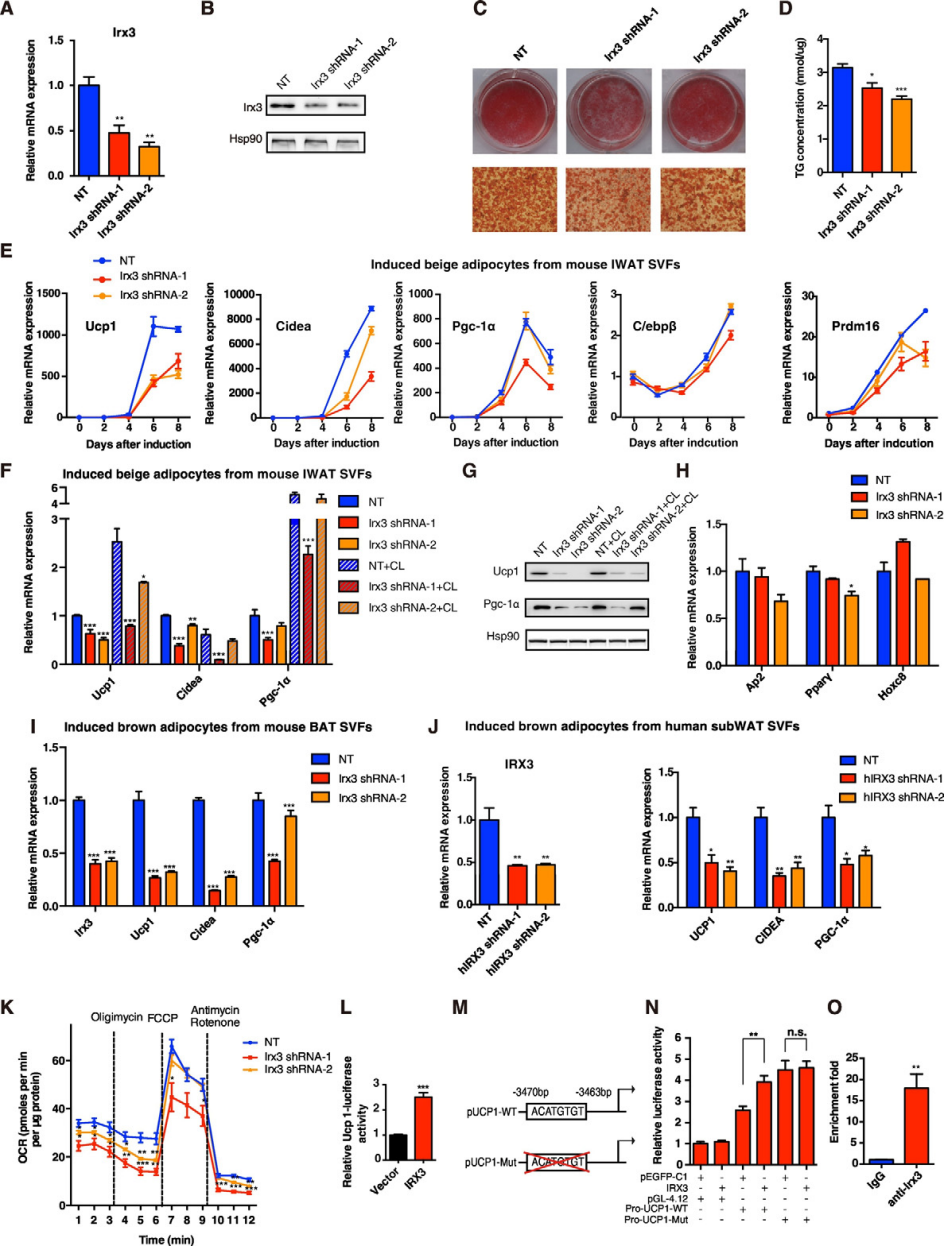
Knockdown of *Irx3* repressed the differentiation of SVFs toward beige adipocytes in vitro. (A–B) (A) Relative mRNA and (B) protein levels of *Irx3* in SVFs from IWAT were efficiently knocked down after a 3-day infection of mouse *Irx3* lentiviral shRNA (*n* = 3 for each group). (C–D) (C) Oil Red O staining and (D) TG concentration of the mature beige adipocytes under eight-day differentiation (*n* = 6 for each group). (E) Relative mRNA expression of *Ucp1*, *Cidea*, *Pgc-1α*, *C/ebpβ*, and *Prdm16* at different time points during beige adipocyte differentiation from IWAT SVFs (*n* = 3–4 for different groups). (F–G) (F) Relative mRNA (*n* = 4) and (G) protein expression of brown adipocyte marker genes of the induced beige adipocytes from mouse IWAT SVTs for eight days with the infection of mouse *Irx3* lentiviral shRNA, with or without CL-316,243 activation. (H) Relative mRNA expression of adipocyte differentiation-related genes in beige adipocytes (*n* = 4). (I) Relative mRNA expression of the indicated genes in the induced brown adipocytes from mouse BAT SVFs for eight days, with the infection of mouse *Irx3* lentiviral shRNA (*n* = 4). (J) Relative mRNA levels of *IRX3* in SVFs from human sWAT after a 3-day infection of human *IRX3* lentiviral shRNA (left), and the mRNA levels of brown adipocyte marker genes in the SVFs under 2-day differentiation (*n* = 4). (K) OCR measurement of the beige adipocytes under 5-day differentiation, with the infection of mouse *Irx3* lentiviral shRNA (*n* = 5). (L–N) (L) *Ucp1* promoter-luciferase reporter activity was measured in HEK293T cells transfected with pEGFP-C1 vector or *IRX3* for 48 h. (M) Schematic diagram of the mutant mouse *Ucp1* promoter deleting ACATGTGT among the − 3470 to − 3463 bp region proximal to the TSS of the *Ucp1* gene. (N) Transcriptional activity of wild-type or mutant *Ucp1* promoter was measured with IRX3 overexpression. (O) qPCR of *Ucp1* promoter binding region which was recruited by Irx3 antibody in 8-day induced beige adipocytes, as analyzed by ChIP. Fold enrichment of *Ucp1* promoter was given (*n* = 3). For the measurement of luciferase activity, cells were seeded on 24-well plate and transfected with 800 ng pEGFP-C1 vector or IRX3, 200 ng *Ucp1* promoter construct, and 1 ng pRL-SV40, followed by the harvest for luciferase activity assessment using a dual-luciferase reporter assay system (Promega). Luciferase activity was corrected for Renilla luciferase activity (*n* = 3–4 for each group). For qPCR data, mRNA expression was normalized to *36b4* for mouse genes and *βACTIN* for human genes. SVF cells were isolated from IWAT and BAT of C57BL/6J mice or human sWAT wherever mentioned, and *IRX3* shRNA were introduced to cells 24 h after seeding. Data were presented as mean ± s.e.m. *P < 0.05, **P < 0.01, ***P < 0.001. The results are representative of at least three independent experiments. NT, no targeting.

To avoid the non-specific or compensatory influence of other tissues in vivo, we next examined the cell-autonomous effects of *Irx3* on the differentiation of beige/brown adipocytes from preadipocytes in SVFs of IWAT and BAT in vitro, respectively. We efficiently knocked down Irx3 expression in SVFs with lentiviral *Irx3* shRNA (Fig. 3A-B) and found that *Irx3* reduction led to less lipid accumulation after beige adipocyte differentiation as determined by Oil Red O staining (Fig. 3C) and triglyceride quantification (Fig. 3D). Furthermore, *Irx3* knockdown repressed the expression of *Prdm16* at early stage of differentiation and markedly inhibited terminal brown adipocyte markers *Ucp1*, *Cidea* and *Pgc-1α* at late stages (Fig. 3E). Of note, *Ucp1*, which is the specific marker for brown adipocytes and functions in uncoupling oxidative metabolism from ATP production (Shabalina et al., 2013), was robustly reduced in beige adipocytes with *Irx3* knockdown, which was more obvious under CL-316,243 treatment (Fig. 3F). *Pgc-1α*, a transcriptional co-regulator of mitochondrial biogenesis (Liang and Ward, 2006), and *Cidea*, which promoted lipolysis and thermogenesis, were also significantly decreased in beige adipocytes with *Irx3* knockdown compared to the controls (Fig. 3F). Besides, protein levels of Ucp1 and Pgc-1α showed similar changes upon Irx3 knockdown (Fig. 3G). Adipocyte differentiation-related genes, *Ap2* and *Pparγ*, and white adipocyte-specific marker *Hoxc8* showed no or mild changes with *Irx3* knockdown (Fig. 3H). Other white adipocyte makers such as *Glut4*, *Serpina3k* and *Wdnm1-like*, were not significantly inhibited by *Irx3* knockdown, and the latter two were rather increased (Supplementary Fig. 2). Furthermore, *Irx3* knockdown also inhibited *Ucp1*, *Cidea* and *Pgc-1α* expression in differentiated brown adipocytes from SVFs in classical BAT (Fig. 3I). More importantly, *IRX3* repression significantly inhibited human beige adipocyte program, reflected by reduced *UCP1*, *CIDEA and PGC-1α* expression in differentiated human SVFs (Fig. 3J). These results together suggested that IRX3 knockdown inhibited the browning program from preadipocytes in WATs of both mice and humans. Beige adipocyte functions in dissipating heat by maintaining a high mitochondrial OCR. Accordingly, measurement of the oxygen respiration ability of the fully differentiated beige adipocytes showed that both basal and uncoupled respiration rates were significantly decreased with or without normalization when *Irx3* was reduced (Fig. 3K and Supplementary Fig. 3A). In addition, OCR measurement using 2% fatty acid-free BSA obtained similar results (Supplementary Fig. 3B). When normalized all OCR data to basal OCR, the relative uncoupled OCR to basal respiration also showed decreased trend by Irx3 knockdown, although not reaching statistical significance (Supplementary Fig. 3C). Collectively, our findings suggested that IRX3 deficiency repressed the beige/brown adipocytes induction from preadipocytes, indicating the promoting effects of *Irx3/IRX3* on the browning program.

### 3.4 *IRX3* Overexpression Increased *UCP1* Transcriptional Activity

*IRX3* is a transcription factor and we found that *IRX3* knockdown significantly repressed *UCP1* expression in the beige adipocytes, based on which we further explored whether *IRX3* could directly regulate the transcriptional activity of *UCP1* promoter. Previous studies have demonstrated that *IRX3* exerts its transcriptional activity by specifically binding to the motif of ACAnnTGT (Bilioni et al., 2005), which is also presented as ACATGTGT among the − 3470 to − 3463 bp region proximal to the TSS of the mouse *Ucp1* gene. Significantly, *IRX3* overexpression increased the transcriptional activity of *Ucp1* promoter containing this motif in a luciferase reporter assay in HEK293T cells (Fig. 3L). Further, we constructed the mutant *Ucp1* promoter plasmids with IRX3 binding motif deletion as indicated in Fig. 3M, which abolished IRX3-enhanced *Ucp1* promoter activity (Fig. 3N), while point mutations (ACATGTGT replaced with ATATGTAT) (Bilioni et al., 2005) in *IRX3* binding motif of *Ucp1* promoter did not abolish IRX3’s activating effects on *Ucp1* promoter activity (Supplementary Fig. 4A). In addition, we also constructed human *UCP1* promoter containing five putative IRX3 binding regions (ACAnnTGT) and five corresponding point mutations with the sequence from ACAnnTGT to ATAnnTAT. Consistently, we found that *IRX3* overexpression also promoted human *UCP1* promoter activity while point mutations did not abolish IRX3’s activating effects on *UCP1* promoter (Supplementary Fig. 4B). To further examine the direct binding effects of Irx3 on *Ucp1* promoter, we conducted ChIP assay and found that Irx3 bound to the putative binding motif on the *Ucp1* promoter in the induced mature beige cells (Fig. 3O), strengthening the result that the transcriptional activity of *UCP1* promoter was enhanced by *IRX3*. These results suggested that *IRX3* promoted *UCP1* transcriptional activity at least in part through the ACATGTGT binding site.

### 3.5 The Association Between IRX3 and Human Obesity

**Fig. 4:**
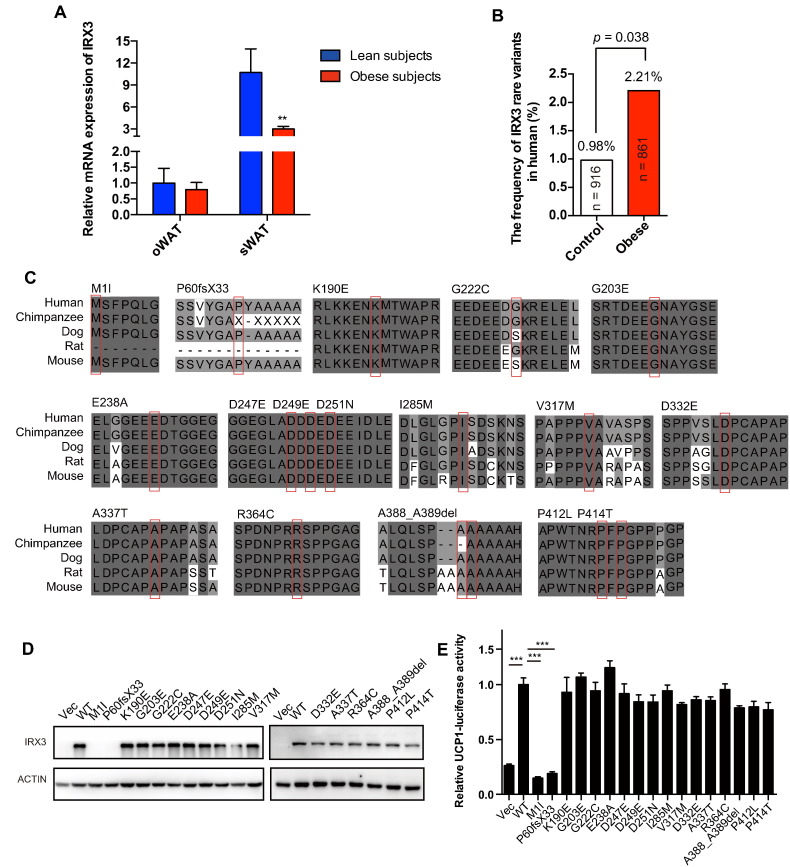
The association of *IRX3* and human obesity. (A) Relative mRNA levels of *IRX3* in oWAT or sWAT from normal weight (*n* = 9) and obese subjects (*n* = 21) measured by qPCR. Gene expression was normalized to *βACTIN*. (B) Comparison of the frequency of the *IRX3* rare variants in normal weight subjects and obese patients. (C) Sequence conservation analysis of the *IRX3* orthologs related to variants identified in obese subjects and controls. Mutant sites are shown with a red box. Dark gray represents residues that are completely conserved, mild gray partially conserved, and light gray shows similar residues. (D) Protein expression levels of WT and mutant IRX3 in HEK293T cells. GFP antibody was used to indicate IRX3, and actin antibody served as a loading control. (E) *Ucp1* promoter-luciferase reporter activity was measured in HEK293T cells transfected with pEGFP-C1 vector, WT or mutant IRX3 for 48 h (*n* = 3). Normalized luciferase activities are shown as fold change and compared to WT IRX3 (*n* = 4). oWAT, omental white adipose tissue. sWAT, subcutaneous white adipose tissue. Data were presented as mean ± s.e.m. *P < 0.05, **P < 0.01, ***P < 0.001. The results are representative of at least three independent experiments.

Taken together, we have identified the promoting effects of *IRX3* on the browning program. To further explore the association of *IRX3* with human obesity, we examined *IRX3* expression in human adipose tissues and detected significantly reduced *IRX3* mRNA levels in the sWAT of obese subjects compared to those of non-obese individuals (Fig. 4A). Moreover, we evaluated the effects of *IRX3* variants on human obesity in a two-stage case-control study including 227 young obese subjects (BMI, 35.1–61.7 kg/m^2^) and 219 lean controls (BMI, 17.5–23.0 kg/m^2^) in the first stage; and 634 young obese subjects (BMI, 30.0–63.3 kg/m^2^) and 697 lean control subjects (BMI, 17.5–23.0 kg/m^2^) in the second stage (Table 1). Stage one identified 5 cases carrying rare nonsynonymous *IRX3* variants with a MAF < 1% but none in the controls; and stage two screened 11 variants from 14 obese subjects and 9 variants from lean subjects. In total, 19 obese patients and 9 lean subjects (2.21% vs. 0.98%) had rare nonsynonymous *IRX3* variants (*P* = 0.038; OR = 2.27; 95% CI, 1.02–5.05) (Fig. 4B), indicating that rare nonsynonymous variants of *IRX3* were associated with the development of human obesity.

**Table 1:** Rare *IRX3* variants were enriched in young obese subjects in comparison to lean controls.

	***N*****total (male)**	**Age (yrs)**		**BMI (kg/m**^**2**^**)**		**Rare*****IRX3*****variant**		
		**Median (inter-quartile range)**	**Range**	**Median(inter-quartile range)**	**Range**	***N*****(%)**	**OR (95% CI)**	***P*****value**
**Stage 1**								
Cases	227 (98)	21 (18, 25)	13.0–30.0	38.4 (36.8, 41.2)	35.0–61.7	5 (2.20)	–	0.061
Controls	219 (92)	38.4 (36.8, 41.2)	13.0–30.0	20.4 (19.5, 21.6)	17–23.0	0 (0)		
**Stage 2**								
Cases	634 (262)	22 (18, 26)	12.0–30.0	34.6 (32.9, 37.3)	30.0–63.3	14 (2.21)	1.73 (0.74, 4.02)	0.20
Controls	697 (244)	25 (22, 28)	14.0–58.0	19.5 (18.3, 21.0)	17.5–23.0	9 (1.29)		
**Combined**								
Cases	861 (360)	22 (18, 25)	12.0–30.0	35.9 (33.6, 38.9)	30.0–63.3	19 (2.21)	2.27 (1.02, 5.05)	0.038
Controls	916 (336)	24 (21, 27)	13.0–58.0	19.8 (18.6, 1.1)	17.5–23.0	9 (0.98)		

N, sample size; Yrs, years; BMI, body mass index; OR, odds ratio; CI, confidence interval.

*P* values were calculated using χ^2^ tests.

A total of 17 rare nonsynonymous variants in heterozygous form were identified in the studied cohort (Table 2). Most of these rare *IRX3* variants were highly conserved among various species, implying the functional importance for the IRX3 protein (Fig. 4C). Some of the variants were predicted to be damaged or possibly damaged by MutationTaster, PolyPhen-2, and SIFT (Table 2). To better validate the function of these variants, we further conducted functional examination of the nonsynonymous variants. We found that two variants, M1I and P60fsX33, markedly changed the protein expression, while no obvious alteration in expression of other variants was observed (Fig. 4D and Supplementary Fig. 5). More importantly, M1I and P60fsX33 mutants markedly eliminated wild-type *IRX3*-induced *Ucp1* transcriptional activity (Fig. 4E), confirming their functional damage. This result suggested that the genetic defects of *IRX3* could possibly repress the browning program in humans at least in part by regulating *UCP1* transcriptional activity to increase the risk of obesity. These findings together suggested the involvement of *IRX3* deficiency in human obesity.

**Table 2:** 17 rare *IRX3* variants identified in young obese subjects and lean controls.

**Position**^a^	**Nucleotide chang**e^b^	**Amino acid change**^b^	**State**	**Cases**	**Controls**	**BMI of cases (kg/m**^**2**^**)**	**BMI of controls (kg/m**^**2**^**)**	**Mutation taster**^#^	**SIFT**^§^	**PolyPhen2**^‡^
chr16:54319960	c.G3 > A	p.M1I	Het	1	0	41.7	–	Deleterious	Deleterious	Possibly damaging
chr16:54319787-54319788	c.175_176delGC	p.P60fsX33	Het	0	1	–	21.6	–	–	–
chr16:54319225	c.568A > G	p.K190E	Het	0	1	–	20.7	Damaging	Damaging	Probably damaging
chr16:54319185	c.608G > A	p.G203E	Het	1	0	36.3	–	Damaging	Damaging	Probably damaging
chr16:54319129	c.664G > T	p.G222C	Het	1	0	38.7	–	Damaging	Damaging	Benign
chr16:54319080	c.713A > C	p.E238A	Het	4	1	34.2, 36.9, 37.0, 45.3	18.2	Tolerated	Tolerated	Benign
chr16:54319052	c.741C > G	p.D247E	Het	2	1	30.8, 32.7	21.2	Tolerated	Tolerated	Benign
chr16:54319046	c.747C > G	p.D249E	Het	2	0	37.3, 39.4	–	Tolerated	Tolerated	Benign
chr16:54319042	c.751G > A	p.D251N	Het	1	1	41.1	20.3	Damaging	Damaging	Possibly damaging
chr16:54318938	c.855T > G	p.I285M	Het	1	0	42.6	–	Tolerated	Tolerated	Benign
chr16:54318844	c.949G > A	p.V317 M	Het	0	1	–	21.1	Tolerated	Tolerated	Benign
chr16:54318797	c.996C > A	p.D332E	Het	0	1	–	21.6	Tolerated	Tolerated	Benign
chr16:54318784	c.1009G > A	p.A337T	Het	0	1	–	19.9	Tolerated	Tolerated	Benign
chr16:54318703	c.1090C > T	p.R364C	Het	1	0	40.6	–	Damaging	Damaging	Probably damaging
chr16:54318631-54318626	c.1162_1167delGCCGCC	p.A388_A389del	Het	0	1	–	21.8	–	–	–
chr16:54318558	c.1235C > T	p.P412L	Het	3	0	34.2, 37.7, 38.9	–	Damaging	Damaging	Possibly damaging
chr16:54318553	c.1240C > A	p.P414T	Het	2	0	33.8, 39.1	–	Tolerated	Tolerated	Benign

Het, heterozygous; BMI, body mass index. Functional prediction was conducted by Mutation taster^#^ (http://mutationtaster.org), SIFT^§^ (http://sift.jcvi.org), and PolyPhen2^‡^ (http://genetics.bwh.harvard.edu/pph2/).

a NCBI Build 37.

b Variations are based on RefSeq records NM_024336.2 and NP_077312.2.

## 4 Discussion

In this study, we showed that IRX3/Irx3 expression was induced in the browning process of WATs in both humans and mice, and its knockdown led to noticeably impaired ability of beige/brown adipocytes program in vitro, possibly by inhibiting *Ucp1* promoter transcriptional activity. We further identified the association between rare nonsynonymous *IRX3* variants with human obesity risk. Taken together, we demonstrated that *IRX3* played a promoting role in the browning of WAT and its rare variants are associated with human obesity.

Weight loss by increasing energy expenditure is an attractive therapeutic approach for obesity and its complications, especially since the identification of heat-dissipating brown adipocytes in human adults (Cypess et al., 2009, 2013; Lidell et al., 2013). The present study demonstrated the effects of *IRX3* on the browning process by the following evidence:﻿ (1) ﻿IRX3/Irx3 reduction by shRNA in vitro dramatically inhibited the browning program and energy expenditure of beige adipocytes, as reflected by the reduced UCP1 levels and oxygen consumption ability, which is a key mechanism for the development of obesity (Wang et al., 2013; Zhang et al., 2014; Lee et al., 2014); ﻿﻿(2) IRX3 significantly activated *UCP1* transcriptional activity; (3) two loss-of-function *IRX3* mutants identified in human largely abolished this activating effect. This evidence well documented the direct promoting effects of IRX3 on the browning program. Consistently, IRX3/Irx3 expression was induced during the browning process of WAT in humans (pheochromocytoma patients) and β3-adrenergic agonist-stimulated mice, supporting the physiological role of *IRX3* in browning. Irx3 expression was also increased during the time course of beige/brown adipocyte differentiation, supporting the promoting effects of IRX3/Irx3 on browning program. Obviously, this conclusion was opposite to previous reports regarding IRX3’s inhibiting effects on browning in mouse models (Smemo et al., 2014). By using global *Irx3*-knockout mice and hypothalamus-conditional dominant negative *Irx3* transgenic mice with non-C57BL/6J background strains, Smemo et al. found that both mouse strains showed increased *Ucp1* expression in perigonadal WAT, indicating its inhibiting role in the browning process of WAT (Smemo et al., 2014). However, another study by Claussnitzer et al. reported that *IRX3* knockdown increased *UCP1* expression only in human preadipocytes with *FTO* risk allele but did not show any effect on *UCP1* expression in human cells with *FTO* wild-type allele (Claussnitzer et al., 2015), indicating that *IRX3* may inhibit browning dependent on certain genetic background. Besides, we showed that IRX3 knockdown significantly inhibited human beige adipocyte program. Whether the discrepancy between our and Claussnitzer's study is due to the genetic background difference like Caucasian versus Chinese, or due to preadipocyte characterization, for instance, the SVFs from adipose tissues in underaged in our study versus SVFs from adults in Claussnitzer's study, could not be excluded. The detailed underlying mechanism is largely unknown and certainly will be worth exploring in future. Investigation of the effect of Irx3 on the browning program in vivo using the adipose tissue specific knockout mouse model which is different from the dominant negative *Irx3* overexpressed model, as used in previous studies Smemo et al., 2014, and with different genetic background such as C57BL/6J strains might give more information regarding the discrepancy. Taken together, these findings may suggest that IRX3 plays a crucial regulatory role in the browning program possibly based on the specific genetic background, and genetic-based stratification may be needed to determine the effectivity of anti-obesity intervention by targeting *IRX3*.

It is widely known that the browning process is characterized by the induction of *UCP1*, the uncoupling protein specialized in thermogenesis of mitochondria. A few transcriptional factors such as *PPARγ* are required for the commitment and differentiation of brown-like adipocytes by targeting *UCP1* promoters. Although early studies focused on the regulating region of the *UCP1* promoter located ~ 2.5 kb proximal to the TSS, Kong et al found that the transcription factor *IRF4* could mediate the transcriptional activity of the *UCP1* promoter outside this region (Kong et al., 2014). In addition, studies from the ENCODE consortium indicated that the entire 10 kb sequence upstream to *UCP1's* TSS could also be of function (https://genome.ucsc.edu/ENCODE/dataSummaryMouse.html). Here, we demonstrated that the *IRX3*-specific binding site ACATGTGT was located in − 3470 ~ – 3463 bp of *UCP1's* TSS, and deletion of this sequence ablated transcriptional activation by *IRX3*. Besides, ChIP assay further identified that Irx3 directly bound to this motif of *Ucp1* promoter, providing the evidence that Irx3 could interact with the *Ucp1* promoter to increase its transcriptional activity and might regulate thermogenesis as a novel transcriptional factor. It is also worth mentioning whether IRX3 also recruited PGC-1α or other co-activators to promote *UCP1* transcription is largely unknown and warranted further investigation. In addition, we also found potential IRX3 binding site on the promoter of *Cidea*, another affected gene by IRX3. The transcriptional regulation of Cidea by IRX3 remained to be determined in future study. Another point that we want to mention is that deletion of the whole IRX3-binding domain in *UCP1* promoter showed increased luciferase activity than wild-type *UCP1* promoter, while point mutations in this domain did not increase *UCP1* promoter activity, either did not affect IRX3’s promoting effects on *UCP1* promoter activity (Supplementary Fig. 4A). These data suggested that the whole IRX3 binding domain might possess an inhibitory effect on *UCP1* transcription. We speculate that there might be an inhibitory factor to competitively bind to this *IRX3* binding domain to play an inhibitory role on *UCP1* transcription activity. Overexpression of IRX3 increased wild-type *UCP1* promoter activity but not the binding motif-deleted *UCP1* promoter. This result suggested that IRX3 might release the inhibitive effects of this domain on *UCP1* transcriptional activity. Taken together, IRX3 might be one activating component in a transcription machinery complex for induction of browning. Detailed mechanism may need more investigations in future study.

*FTO* and *IRX3* could influence obesity organically (Claussnitzer et al., 2015). Although the strong association between *FTO* variations and obesity has been continuously reported, the genetic defects of the *IRX3* gene per se on obesity have not been clarified until now. As the “missing heritability” for complicated disease is partly attributed to low-frequency and rare variants in the candidate genes with a relatively moderate to large effect size (Liu and Song, 2010; Eichler et al., 2010), it seems that targeted resequencing to identify such variants followed by functional validation is an effective strategy to uncover the genetic basis for complex disease including human obesity. In line with this strategy, we have previously identified that a gain-of-function variant in *LGR4* (Wang et al., 2013; Zou et al., 2017) and rare loss-of-function variants in *NPC1* (Liu et al., 2017) predispose to obesity. In this study, we first revealed the genetic structure of rare variants of *IRX3* in relation to obesity. By targeted sequencing of *IRX3* coding regions in a relatively large number of young obese and lean subjects, we found that rare nonsynonymous *IRX3* variants were enriched in the obese population. Moreover, two nonsense mutants significantly abolished the promoting effect of IRX3 on *UCP1* transcriptional activity. These findings together may suggest that loss-of-function mutation in *IRX3* may increase human obesity risk by inhibiting browning capacity. More human studies were warranted to confirm this point. We also found significantly lower expression of *IRX3* in the subcutaneous fat of obese subjects, which was consistent with two previous studies (Dankel et al., 2010; Landgraf et al., 2016), further supporting the involvement of *IRX3* in human obesity. In addition, we want to mention that the two mutations were respectively detected in an obese subject and a normal-weight subject, which suggested that genetic heterozygosity for a severely dysfunctional *IRX3* allele did not determine body weight following a classic Mendelian model, as seen in monogenic genes for obesity such as *POMC* (Flickinger and Salz, 1994; Krude et al., 1998), *MC4R* (Vaisse et al., 1998; Yeo et al., 1998), and *LEP* (Montague et al., 1997; Echwald et al., 1997). As such, *IRX3* might interact with other obesity-related genes or environmental factors in a complicated manner to determine the penetrance for obesity, although its nonsynonymous variants increased the risk of obesity in humans. A larger-scale case-control replication study or population-based study with a highly homogeneous environmental and genetic background is needed for further investigation.

In summary, our cellular and genetic evidence supported a directly promoting effect of *IRX3* on the browning program of white adipocytes by regulating *UCP1* transcription and provided a new view for the association of rare variants in *IRX3*, as one candidate target of *FTO* variants, with human obesity risk.

## Funding Sources

This research was supported by grants from the National Natural Science Foundation of China (81522011, 81570757, 81570758, 81370963, 81370934 and 81370949), National Basic Research Program of China (2015CB553601), Foundation for Innovative Research Groups of the National Natural Science Foundation of China (81621061), National International Science Cooperation Foundation (2015DFA30560), Shanghai Municipal Education Commission-Gaofeng Clinical Medicine Grant Support (20161306, 20171903), Shanghai Rising-Star Program (17QA1403300), Shanghai Municipal Commission of Health and Family Planning (2017YQ002).

## Conflict of Interest

No potential conflicts of interest relevant to this article were reported.

## Author Contributions

J.W., W.W., G.N., and W.G. conceived the project and designed the experiments. Y.Z. and P.L. carried out most of the experiments. N.C. assisted in some experiments. Y. Z., J.W. and R.L. wrote the paper. J.S., W.L., M.Y., S.Z., M.C., Y.S., and T.N. recruited obese and control participants in stages one. A.G., Q.C., T.N., and Q.M. provided rodent biological samples for association analysis. X.Y., J.J., X.D., and B.S. provided white adipose samples of obese subjects who received sleeve gastrectomy surgery for weight loss. B.Y. provided subcutaneous adipose samples of subject. W.G., Y.F.Z., J.H., W.W., and G.N. designed and set up the GOCY study. Y.S. assisted with statistical analysis. S.L. and S·K contributed valuable comments and advice on the manuscript. G.N., W.W., W.G., and J.W. are the guarantors of this work and, as such, had full access to all the data in the study and took responsibility for the integrity of the data and the accuracy of the data analysis.

## Appendix A Supplementary data

Supplementary material Image 1
