# Supplementary material for: IRX3 Promotes the Browning of White Adipocytes and Its Rare Variants are Associated with Human Obesity Risk
Source: eBioMedicine. 2017 Sep 13;24:64–75. doi: 10.1016/j.ebiom.2017.09.010 (PMC5652024; doi:10.1016/j.ebiom.2017.09.010)
Supplement: Supplementary file 1 — Supplementary material [file mmc1.doc]

**Supplementary Materials**

**Table of contents:**

Table S1. Primers used in qPCR.

Table S2. Clinical characteristics of obese subjects and lean controls whose fat tissues were collected during surgery.

Table S3. Primers used for genomic PCR and Sanger sequencing of *IRX3*.

Figure S1. Protein levels of Ucp1 and Irx3 in IWAT from mice under cold stress (4°C) for 1 week and at 25°C

Figure S2. Relative mRNA expression of white adipocyte marker genes in the beige adipocytes under 8-day differentiation.

Figure S3. OCR measurement of the beige adipocytes under 5-day differentiation.

Figure S4. Transcriptional activity of wild-type or mutant *UCP1* promoter with IRX3 overexpression.

Figure S5. Protein expression levels of wild-type IRX3, M1I, and P60fsX33 in HEK293T cells.

| **Table S1. Primers used in qPCR** | | | | | | | | |
| --- | --- | --- | --- | --- | --- | --- | --- | --- |
| Primer name | | Primer sequence | | |  | | | |
| h βactin -F | | TGACGTGGACATCCGCAAAG | | |  | | | |
| h βactin -R | | CTGGAAGGTGGACAGCGAGG | | |  | | | |
| h IRX3-F | | AAAAGTTACTCAAGACAGCTTTCCA | | |  | | | |
| h IRX3-R | | GGATGAGGAGAGAGCCGATA | | |  | | | |
| h UCP1-F | | AGGTCCAAGGTGAATGCCC | | |  | | | |
| h UCP1-R | | TTACCACAGCGGTGATTGTTC | | |  | | | |
| h PGC-1α-F | | TCTGAGTCTGTATGGAGTGACAT | | |  | | | |
| h PGC-1α-R | | CCAAGTCGTTCACATCTAGTTCA | | |  | | | |
| h CIDEA-F | | GATGCCCTCGTCATCGCTAC | | |  | | | |
| h CIDEA-R | | GCGTGTTGTCTCCCAAGGTC | | |  | | | |
| m 36b4-F | | GAAACTGCTGCCTCACATCCG | | |  | | | |
| m 36b4-R | | GCTGGCACAGTGACCTCACACG | | |  | | | |
| m Irx3-F | | GGCAATGCTTATGGGAGCGA | | |  | | | |
| m Irx3-R | | CGCTGTCTAAGTTTTCCAAATCG | | |  | | | |
| m Ucp1-F | | AGGCTTCCAGTACCATTAGGT | | |  | | | |
| m Ucp1-R | | CTGAGTGAGGCAAAGCTGATTT | | |  | | | |
| m Pgc-1α-F | | AGCCGTGACCACTGACAACGAG | | |  | | | |
| m Pgc-1α-R | | GCTGCATGGTTCTGAGTGCTAAG | | |  | | | |
| m Cidea-F | | TGCTCTTCTGTATCGCCCAGT | | |  | | | |
| m Cidea-R | | GCCGTGTTAAGGAATCTGCTG | | |  | | | |
| m Hoxc8-F | | GTCTCCCAGCCTCATGTTTC | | |  | | | |
| m Hoxc8-R | | TCTGATACCGGCTGTAAGTTTGT | | |  | | | |
| m Ap2-F | | ACACCGAGATTTCCTTCA AACTG | | |  | | | |
| m Ap2-R | | CCATCTAGGGTTATGATGCTCTTCA | | |  | | | |
| m Pparγ2-F | | GCATGGTGCCTTCGCTGA | | |  | | | |
| m Pparγ2-R | | TGGCATCTCTGTGTCAACCATG | | |  | | | |
| m Dio2-F | | CAGTGTGGTGCACGTCTCCAATC | | |  | | | |
| m Dio2-R | | TGAACCAAAGTTGACCACCAG | | |  | | | |
| m Leptin-F | | GAGACCCCTGTGTCGGTTC | | |  | | | |
| m Leptin-R | | CTGCGTGTGTGAAATGTCATTG | | |  | | | |
| m Cebp/β-F | | CAAGCTGAGCGACGAGTACA | | |  | | | |
| m Cebp/β-R | | AGCTGCTCCACCTTCTTCTG | | |  | | | |
| m Prdm16-F | | CCACCAGCGAGGACTTCAC | | |  | | | |
| m Prdm16-R | | GGAGGACTCTCGTAGCTCGAA | | |  | | | |
| m Wdnm1-like1-F | | TGTGGGCCAGAGGAACAATG | | |  | | | |
| m Wdnm1-like1-R | | ACTCCACTGTGCTGCTTGTA | | |  | | | |
| m SerpinA3k-F | | GGCTGAAGGCAAAGTCAGTGT | | |  | | | |
| m SerpinA3k-R | | TGGAATCTGTCCTGCTGTCCT | | |  | | | |
| m Glut4-1-F | | GTGACTGGAACACTGGTCCTA | | |  | | | |
| m Glut4-1-R | | CCAGCCACGTTGCATTGTAG | | |  | | | |
| M, mouse; H, human. F, Forward primer; R, Reward primer. | | | | |  | | | |
| **Table S2. Clinical characteristics of obese subjects and lean controls whose fat tissues were collected during surgery.** | | | | | | | | |
| **Characteristics** | **Cases** | | **Controls** | ***P* Value** | |  | |  |
| **N total (Male)** | 21 (9) | | 9 (4) | 0.75 | |  | |  |
| **Age (yrs)** |  | |  | <0.0001 | |  | |  |
| Mean (SD) | 31.1 (8.5) | | 50.9 (10.7) |  | |  | |  |
| Range | 20.0 - 55.0 | | 28.0 - 62.0 |  | |  | |  |
| **BMI (kg/m^2^)** |  | |  | <0.0001 | |  | |  |
| Mean (SD) | 46.3 (7.9) | | 24.6 (2.0) |  | |  | |  |
| Range | 37.0 - 68.7 | | 21.5 - 27.0 |  | |  | |  |
| N, sample size. Yrs, years. BMI, body mass index.  *P* values were calculated using Student’s *t*-test | | | | | | |  | |

| **Table S3. Primers used for genomic PCR and Sanger sequencing of *IRX3*.** | | | |
| --- | --- | --- | --- |
| **Exon** | **Primer name** | **Primer sequence** | **PCR products (bp)** |
| Exon 1 | E1-F | CCCGTAGAAATGTCAATCAGAGC | 729 |
|  | E1-R | CTGTCCTTCAGCTCATACTGCG |  |
| Exon 2 | E2-1F | CTTCCCGCAGCTGGTAAGAG | 671 |
|  | E2-1R | TCGTCCTCGTCGTCGTCAGC |  |
| Exon 2 | E2-2F | CAGGTGTCCACCTGGTTCGC | 723 |
|  | E2-2R | AAACGGCCGGTTGGTCCAAG |  |
| Exon 3 | E3-F | CCCGTGAGCCTGGACCCCTG | 897 |
|  | E3-R | GCTTCCCCTAGAAGGTACAAGCG |  |
| Exon 4 | E4-1F | GACATAATTTTACATTGCGACACC | 681 |
|  | E4-1R | ACTCGGTCCCGATTCGTCTC |  |
| Exon 4 | E4-2F | CATTGCGACACCTTCCTGAC | 505 |
|  | E4-2R | ACTTTCTCGTACAATTTGGTTCAC |  |
| F, Forward primer; R, Reward primer. | | | |

**Figure S1. Protein levels of Ucp1 and Irx3 in IWAT from mice under cold stress (4°C) for 1 week and at 25°C (n = 5).**

**Figure S2. Relative mRNA expression of white adipocytes marker genes in the induced beige adipocytes from preadipocytes isolated from IWAT.** The expression of *Irx3* was knocked down in SVFs isolated from IWAT by two *Irx3* lentiviral shRNAs. The cells were induced to beige adipocytes under 8-day differentiation. mRNA levels were determined by realtime PCR and normalized to 36b4 (n = 4). Data were presented as mean ± s.e.m. * *P* <0.05, ***P* < 0.01, ****P* < 0.001.

**Figure S3. OCR measurement of the beige adipocytes under 5-day differentiation.** The expression of *Irx3* was knocked down in SVFs isolated from IWAT by two mouse *Irx3* lentiviral shRNAs. Once reaching confluence, the cells were induced to beige adipocytes for five days. Basal respiration, basal proton leak respiration (after oligomycin injection), maximal respiratory capacity (FCCP) and non-mitochondrial respiration (antimycin A, rotenone) relative to basal OCR in the cells without BSA (A) were determined (n = 3-5 for different groups). OCR levels of the cells supplemented with 2% fatty acid-free BSA (B) and the relative OCR to basal value (C) were determined (n = 3-5 for different groups). Data were presented as mean ± s.e.m. * *P* <0.05, ***P* < 0.01, ****P* < 0.001.

**Figure S4. Transcriptional activity of wild-type or mutant *UCP1* promoter in the presence or absence of IRX3.** HEK293T cells were seeded on 24-well plate and transfected with 800ng pEGFP-C1 vector or IRX3, 1ng pRL-TK, 200ng mouse Ucp1 promoter construct (A) or human UCP1 promoter construct (B), followed by harvest for luciferase activity assessment using a dual-luciferase reporter assay system (Promega). Luciferase activity was corrected for Renilla luciferase activity (n = 4). Data were presented as mean ± s.e.m * *P* <0.05, ***P* < 0.01, ****P* < 0.001. The results are representative of at least three independent experiments.

**Figure S5. Protein expression levels of wild-type IRX3, M1I, and P60fsX33 in HEK293T cells.** GFP antibody was used to indicate IRX3, and Actin served as a loading control. The result is representative of at least three independent experiments.
